# Health Economic Analysis of an All-Virtual, At-Home Acute Care Model

**DOI:** 10.1001/jamanetworkopen.2025.17114

**Published:** 2025-06-23

**Authors:** Brad Spellberg, Christopher Lynch, Hal F. Yee, Josh Banerjee

**Affiliations:** 1 Hospital Administration, Los Angeles General Medical Center, Los Angeles, California; 2 Department of Medicine, Los Angeles General Medical Center, Los Angeles, California; 3 Department of Medicine, University of California, San Francisco School of Medicine, San Francisco, California

## Abstract

**Importance:**

An all-virtual, at-home acute care model, called Safer@Home, was found to enable an average 4-day reduction in hospital length of stay. The program is not currently reimbursed.

**Objective:**

To estimate costs and savings associated with the Safer@Home program from a hospital and payer perspective.

**Design, Setting, and Participants:**

This retrospective, economic evaluation analyzed costs associated with the Safer@Home program at a large, academic, public, level I trauma hospital near downtown Los Angeles, California, between September 2022 and August 2023. Patients with 1 of 10 protocolized diagnoses were eligible for the program. Data analysis occurred from January to July 2024.

**Exposure:**

Patients who presented to the hospital and were enrolled in an all-virtual, at-home acute care program called Safer@Home were compared with matched controls with similar diagnoses who received entirely in-hospital care.

**Main Outcome and Measures:**

The primary outcome was estimated net hospital and payer cost with the program vs without. Revenue from third-party payers was compared with hospital variable costs.

**Results:**

A total of 876 patients receiving care in the Safer@Home program (541 male [61.8%]; mean [SD] age, 54 [15 years]; mean [SD] expected mortality, 1.6% [4.7%]; mean [SD] case mix index, 1.27 [0.66]) were compared with 1590 matched control patients (901 male [56.7%]; mean [SD] age, 52 [20] years; mean [SD] expected mortality, 1.9% [5.9%]; mean [SD] case mix index, 1.26 [0.59]). Safer@Home enabled net hospital savings of $5.60 million, calculated as variable costs saved minus revenue lost, for the enrolled patients. Overall savings were due to net savings for Medicaid ($8380 per patient) and unfunded patients ($10 934 per patient), but net losses were due to significant loss of revenue for Medicare (−$4143 per patient) and commercially insured patients (−$25 999 per patien). Modeling demonstrated that revenue based on payer mix, rather than avoided variable hospital costs, was the primary factor of net hospital savings and losses. Absent reimbursement, the program was cost-saving to payers in all modeled scenarios. Creating reimbursement rates of 50% to 60% of hospital costs would enable the program to be cost-saving to both the hospital and payers, across payer mixes.

**Conclusions and Relevance:**

In this economic evaluation study, an all-virtual, at-home acute care program was associated with both hospital and payer savings; however, in the absence of reimbursement, it was only cost-saving to hospitals for Medicaid-funded or uninsured patients. These findings suggest that payer reform is needed to enable program generalization.

## Key Points

**Question:**

Can an all-virtual, at-home acute care program be cost-saving to both hospitals and payers?

**Findings:**

This economic evaluation of 876 patients receiving all-virtual, at-home acute care and 1590 matched controls found that the virtual program was cost-saving (due to avoided variable hospital costs) only for unfunded patients or patients with Medicaid, but was cost-losing for Medicare and commercially insured patients due to substantial lost inpatient revenue. However, a reimbursement schema that paid 50% to 60% of hospital inpatient variable costs would allow the program to be net saving for both hospitals and payers.

**Meaning:**

These findings suggest that current payer rates for inpatient care financially disincentivize development and implementation of novel acute, at-home care models, but a simple reimbursement schema could enable net savings to both hospitals and payers.

## Teaser

This economic evaluation estimates hospital and payer costs associated with an all-virtual, at-home acute care program.

## Introduction

Hospital-at-home care models send staff, durable medical equipment (DME), and therapeutics (eg, intravenous infusions) to patients’ homes to provide care.^1,2,3,4,5^ In the US, Medicare requires in-person evaluations at least twice daily to receive payment for hospital-at-home services. However, dispersing hospital staff to patients’ homes at least twice daily may be infeasible for medical centers that service large geographical and/or urban regions with heavy traffic. In the UK National Health Service, virtual wards at home are increasingly being used in lieu of inpatient care.^6^

Similarly, we developed an all-virtual, at-home acute care model, called Safer@Home, at our large, urban public hospital near downtown Los Angeles, California. The Safer@Home model was based on recent evidence that treatments administered orally are as effective as intravenously, even in patients with acute illnesses that have historically required hospitalization,^7,8,9,10,11,12^ and that acutely required supplemental oxygen via nasal canula can be safely managed at home rather than in hospital.^3,13^ Thus, acutely ill patients enrolled in the program, who otherwise would have required inpatient hospital care, were discharged home with oral or inhalational therapy in lieu of intravenous treatment, and with remote vital sign monitoring and virtual check-ins by health care professionals.

As we have previously published,^14^ during its first year, 876 patients were treated via Safer@Home after enrollment from the emergency department (46%), observation status (8%), urgent care (2%), or the inpatient setting (45%). By comparison, 1590 control patients matched by diagnosis and procedure codes, age, gender, ethnicity, case mix index, and expected mortality, were treated in the inpatient setting. Safer@Home patients had an average of 4 days shorter length of stay than concurrent control patients, with no difference in mortality or 30-day hospital readmission.^14^ Here, we describe the economic impact of the program from both the hospital and payer perspectives.

## Methods

### Study Design, Setting, and Population

This economic evaluation was approved as expedited and with a waiver of informed consent by the University of Southern California institutional review board and follows the Consolidated Health Economic Evaluation Reporting Standards (CHEERS) reporting guideline.^15^ This analysis considered patients who received acute care in the first year of the Safer@Home program, from September 1, 2022, through August 31, 2023, at the Los Angeles General Medical Center, a 676-bed, level I trauma, safety net teaching hospital near downtown Los Angeles. Beginning with the program’s launch, emergency and hospital clinicians were encouraged, but not required, to refer eligible patients to Safer@Home with 1 of 10 protocolized diagnoses (eTable 1 in Supplement 1), or for other conditions if the patient met other general eligibility criteria.^14^ Patients of any age were eligible for Safer@Home if they would have required inpatient care in the absence of the program, were hemodynamically stable, could tolerate and absorb oral or inhalational medications, and had a discharge address and phone number. Safer@Home nurses and hospitalists provided coverage 12 hours a day, 7 days a week, with daily virtual-visits and as-needed in-person evaluations via urgent care.^14^ After the first year of the program, we retrospectively compared patients in the program with matched control patients who received care entirely inpatient for the same clinical conditions.^14^

### Hospital Perspective Economics of the Program

#### Hospital Costs

Hospital variable costs were reduced by shortening inpatient stay compared with control patients. Daily hospital variable costs (average of $2945 per day) were obtained from our finance department and reflect actual inpatient variable costs incurred (rather than charges), averaged over all inpatient encounters at our hospital during the study period. Variable costs included only those costs that could be affected by altering inpatient work volumes and excluding employee salary and benefits, utilities, and overhead.

Safer@Home programmatic fixed costs were incurred due to staffing and DME with which patients were discharged home (eg, pulse oximeters, blood pressure cuffs, or scales for daily weights for patients with congestive heart failure) (eTable 1 in Supplement 1). The program was staffed with 2 full-time equivalent (FTE) registered nurses and one 0.5 FTE hospitalist, via rotating staff.

#### Lost Hospital Revenue

For Medicare-funded patients, lost hospital revenue from avoided admissions or admissions lasting less than 2 midnights was calculated by multiplying the hospital’s base Medicare payment rate by the Medicare severity diagnosis-related group (DRG) relative weight, adding in disproportionate share payments and the daily per diem Medicare rate. For Fee-for-Service (FFS) Medicaid, LA General was reimbursed on a daily flat rate, rather than via DRG payments as some hospitals have switched to. Thus, lost revenue for avoided admissions from FFS Medicaid patients were calculated based on the reimbursable cost per day LA General receives from FFS Medicaid.

LA General in-plan, assigned, managed care Medicaid patients are funded on a per-member, per-month capitated model, and generate no revenue from admission; therefore, no revenue was lost by avoiding such admissions. Similarly for uninsured patients, we presumed no revenue was lost. Lost revenue for out-of-plan Medi-Cal managed care patients, or infrequent commercially insured patients, US Department of Veterans Affairs (VA) and Tricare patients, and others (eg, worker’s compensation) were based on the fixed daily rate the hospital charges each health plan.

### Statistical Analysis

Safer@Home provides a potential cost advantage to payers by reducing inpatient payment to hospitals, while also reducing lost wages and out-of-pocket costs for patients and caregivers. A conservative recent estimate of the average payer billing resulting from 1 hospitalization in the US was approximately $16 900, adjusted for inflation to 2024 by the Consumer Price Index (CPI).^16^ A separate estimate of lost wages and out-of-pocket costs for hospitalized patients and their caregivers was approximately $13 300, again adjusted for inflation.^17^ Finally, urgent care clinic was used to enable rapid outpatient follow-up for Safer@Home patients who needed in person attention as determined by the daily virtual follow up visits. As intended, 30-day follow up urgent care revisits were significantly more common for Safer@Home patients than controls (0.61 vs 0.08 visits per patient). Published costs of urgent care visits in the US have ranged from a low of $213 to a high of $321 in 2024 CPI-adjusted dollars.^18,19,20^

Thus, a reasonable base-case estimate of the combined payer costs of 1 hospitalization with resulting lost wages, out-of-pocket costs, and urgent care follow up visits, was $30 000 ($16 900 for inpatient billing, $13 300 for lost wages and out of pocket costs, and $200 to $300 for an urgent care visit). We modeled payer cost ranges as low as $20 000 and as high as $45 000 per hospitalization.

Based on the reported 75% reduction in length of stay affected by the Safer@Home program,^14^ we modeled a reduction in hospitalization costs by 75% in the base case, with sensitivity analyses ranging from 60% to 90%. Patients are also likely able to return to work earlier, and incur fewer out-of-pocket costs, with the patient at home rather than in the hospital. Nevertheless, because the patients remained acutely ill while at home in Safer@Home, we presumed they still spent the majority of their time unable to work and therefore modeled a recovery of lost wages and out-of-pocket costs of between 15% and 45%, with 30% in the base case.

For quality-of-life (QoL) adjustment, previous studies of patients discharged home with substantial debilitation after receiving critical care or of inpatients with acute illness requiring procedural care have used QoL adjustment scores of 0.6 on a scale where 0 = death and 1 = normal function.^21,22,23,24,25^ We therefore modeled scores of 0.7 or 0.75 for patients still in the hospital, and 0.9 or 0.85 for patients discharged from the hospital in the Safer@Home program.

Statistical analyses were performed from January to July 2024 using Stata software version X (StataCorp). The threshold for statistical significance was a 2-sided *P* < .05.

## Results

### Base Case Hospital Costs and Savings

As previously described, 876 patients (541 male [61.8%]; mean [SD] age, 54 [15 years]; mean [SD] expected mortality, 1.6% [4.7%]; mean [SD] case mix index, 1.27 [0.66]) receiving care in the Safer@Home program were compared with 1590 matched control patients (901 male [56.7%]; mean [SD] age 52 [20] years; mean [SD] expected mortality, 1.9% [5.9%]; mean [SD] case mix index, 1.26 [0.59]).^14^ Fixed costs of operating the program included $518 000 annually for salary and employee benefits for 2 FTE registered nurses and one 0.5 FTE hospitalist. Total DME costs for the 876 patients enrolled over 1 year was $164 547 (eTable 1 in Supplement 1). Thus, total fixed costs were $682 547.

Given the length of stay of the patients in the Safer@Home program compared with control patients treated entirely inpatient, a total of 3504 inpatient bed-days were avoided via the Safer@Home program.^14^ Based on these avoided bed days, the base case estimate for variable hospital cost reduction was $10.31 million, with $4.03 million in revenue lost from avoided admissions (Table 1). Subtracting revenue lost and the fixed DME costs, the net hospital savings in the base case were $5.60 million, or $6392 per patient enrolled in the program (Table 1). Sensitivity analyses comparing length of stay of enrolled patients with those with the same diagnoses cared for in the baseline year prior to program implementation, or with estimated length of stay based on the Safer@Home patients’ all patient refined DRG diagnoses, resulted in estimated net hospital savings of $4.70 million ($5360 per patient) or $6.41 million ($7321 per patient), respectively (Table 1).

**Table 1.** Net Hospital Financial Outcomes: Cost Avoidance vs Lost Revenue and Fixed Costs

Analysis	Insurance, No. (%) (N = 876)	Total inpatient days saved	Total lost revenue, $	Total variable costs avoided $	Total fixed costs of the program, $^a^	Net revenue lost and cost saved, $	Net revenue (cost per case), $
Base case analysis							
Medicare	121 (13.8)	NA	−1 876 585	1 469 533	−94 279	−501 331	−4143
Medicaid	676 (77.2)	NA	−1 670 311	7 861 619	−526 714	5 664 594	8380
Commercial	11 (1.3)	NA	−444 152	166 734	−8571	−285 989	−25 999
Tricare and VA	3 (0.3)	NA	−34 256	48 040	−2337	11 447	3816
Uninsured	65 (7.4)	NA	NA	761 348	−50 646	710 702	10 934
Total	NA	−3504	−4 025 305	10 307 274	−682 547	5 599 422	6392
Sensitivity analysis using baseline control patients^b^	NA	−3125	−3 826 912	9 204 910	−682 547	4 695 451	5360
Sensitivity analysis using APR-DRG predicted LOS	NA	−3701	−3 785 713	10 881 407	−682 547	6 413 147	7321

Abbreviations: APR-DRG, all patient-related, diagnostic-related groups; LOS, length of stay; NA, not applicable; VA, US Department of Veterans Affairs.

^a^ Fixed costs = 2 nursing full-time equivalent and one 0.5 full-time equivalent hospitalist, and $1 million in durable medical equipment.

^b^ Baseline control patients from the year prior to program launch.

Overall, the hospital lost revenue on 316 Safer@Home patients (36.1%) (eTable 2 in Supplement 1). For the remainder, no revenue was lost because the patients were capitated to Los Angeles County Department of Health Services, were uninsured, or had Medicare and stayed in the hospital for more than 2 midnights, resulting in no loss of DRG-based payment. Of the patients for whom any revenue was lost, the majority (224 patients [70.9%]) had some form of Medicaid not capitated to Los Angeles County Department of Health Services, resulting, however, in a minority of lost revenue due to decreased inpatient stay ($1.67 million [41.5%]). In contrast, only 78 patients for whom revenue was lost (24.7%) had Medicare, but those patients accounted for $1.88 million (46.6%) of lost revenue. Similarly, only 11 patients (3.5%) had commercial insurance, but those patients accounted for $0.44 million (11.0%) of lost revenue. When comparing cost savings with revenue lost and fixed costs of the program, the hospital saved a net average of $8380 and $10 934 per Medi-Cal and self-pay patient, respectively, but lost $4143 and $25 999 per Medicare and commercially insurance patient, respectively (Table 1).

### Modeling Hospital Cost and Savings for Different Payer Mixes

Given our hospital’s atypical payer mix, with 84.6% (676 patients) Medicaid and uninsured and only 1.3% (11 patients) commercially insured (Table 1), we modeled the financial impact of the program for a more standard payer mix. According to 2021 analyses of 5800 US hospitals and of the uninsured rate of the general population in the US, approximately 45% of hospital inpatient days across the US were paid via some form of private or commercial insurance, 35% by Medicare, 10% by Medicaid, and 10% by self-pay or uninsured.^26,27^ Given this more typical payer mix and LA General’s daily variable daily cost of hospitalization, enrolling 1000 patients in Safer@Home in the absence of any reimbursement for operating the program would have resulted in a net hospital loss of $11.22 million (Table 2), due to substantial lost revenue from commercially insured patients, and to a lesser extent from Medicare patients.

**Table 2.** Sensitivity Analysis of Payer Mix and Variable Costs on Fiscal Sustainability of Safer@Home for Hospitals in the Absence of Program Reimbursement

Payer	Admissions, %	Hospital net savings or losses for 1000 patients by payer, $				
		Base case daily variable costs ($2945/d)	Variable costs 50% lower ($1473/d)	Variable costs 25% lower ($2209/d)	Variable costs 25% higher ($3681/d)	Variable costs 50% higher ($4418/d)
**Base case payer mix**						
Commercial	1.6^a^	−415 984	−537 245	−476 614	−355 353	−294 723
Medicare	13.8	−571 766	−1 409 764	−990 765	−152 767	266 232
Medicaid	77.2	6 469 033	1 980 002	4 224 517	8 713 548	10 958 064
Uninsured	7.4	809 107	375 725	592 416	1 025 798	1 242 490
Total	100	6 290 571	408 717	3 349 554	9 231 227	12 172 063
**Typical US payer mix** ^26,27^						
Commercial	45	−11 699 547	−15 110 015	−13 404 781	−9 994 314	−8 289 080
Medicare	35	−1 450 131	−3 575 489	−2 512 810	−387 452	675 227
Medicaid	10	837 958	256 477	547 217	1 128 698	1 419 438
Uninsured	10	1 093 388	507 736	800 562	1 386 124	1 679 040
Total	100	−11 218 333	−17 921 291	−14 569 812	−7 866 854	−4 515 375
**One-half of typical commercial and Medicare and triple average Medicaid and self-pay frequency**						
Commercial	22.5	−5 849 774	−7 555 007	−6 702 391	−4 997 157	−4 144 540
Medicare	17.5	−725 066	−1 787 745	−1 256 405	−193 726	337 613
Medicaid	30	3 351 831	1 025 908	2 188 869	4 514 792	5 677 753
Uninsured	30	2 186 776	1 015 472	1 601 124	2 772 428	3 358 080
Total	100	−1 036 233	−7 301 373	−4 168 803	2 096 337	5 228 907
**Maximum commercial payer frequency for net zero hospital savings or losses, %**						
Commercial	NA	29.6	13.1	21.2	38.4	47.7
Uninsured	NA	69.4	86.9	78.8	61.6	52.3

Abbreviation: NA, not applicable.

^a^ Tricare patients grouped with Commercial.

Even halving the typical frequency of commercial- and Medicare-insured patients resulted in a net hospital loss from the program (Table 2). An extreme case analysis in which only 2 payer categories were available—commercial, which resulted in the greatest net losses from the program, and uninsured, which resulted in the greatest net gains⋅for any payer mix involving more than 29.6% of commercially insured patients—resulted in net hospital losses from the program (Table 2).

Payer mix was a significantly greater factor of net savings and losses than daily variable costs. Using the base case payer mix typical of a safety net hospital, dropping hospital variable costs by 50% still resulted in net savings (Table 2). However, using a more typical payer mix, no change in variable costs resulted in net savings from the program. Halving the typical frequency of commercial and Medicare patients enabled cost savings only if variable costs were at least 25% higher than at LA General. Testing the extreme scenario of only commercially insured and uninsured patients, the program could achieve cost neutrality with a frequency of commercially insured patients of 47.7% only if daily inpatient variable costs were 50% higher than at LA General.

### Modeling Reimbursement Rates for Safer@Home Hospital Cost Neutrality

To make the program cost-feasible for hospitals with a typical payer mix, some reimbursement for the program would need to be available. We sought to determine what payment threshold for patients enrolled in the program would enable a hospital to operate Safer@Home at break-even status, where costs saved plus insurance payments were approximately equivalent to lost inpatient revenue plus fixed costs. We imputed Medicaid payments at one-half the reimbursement paid by commercial insurance and Medicare, and did not impute any payment for uninsured patients.

With LA General’s payer mix, no reimbursement was needed to enable hospital cost neutrality (eTable 3 in Supplement 1). Using a more typical payer mix, in which 45% of patients have commercial insurance and 35% have Medicare, $13 198 of reimbursement per patient (one-half of that for Medicaid) was needed to ensure cost neutrality at the LA General daily variable cost rate. The reimbursement threshold for hospital cost neutrality ranged between a low of $5312 if daily variable costs were 50% higher than LA General, to a high of $21 084 if daily variable costs were 50% lower than LA General.

### Payer Cost Implications of Safer@Home

In the base case, Safer@Home decreased payer costs that would have occurred without the program by 55% to 58% (Table 3). QoL–adjusted cost savings were higher (>65%).

**Table 3.** Base Case Payer Savings vs Losses of Safer@Home With and Without Reimbursement^a^

Comparison	Payer costs per case by breakdown of cost without Safer@Home (hospital billing and lost productivity), $ (%)					
	$20 000	$25 000	$30 000	$35 000	$40 000	$45 000
No Safer@Home reimbursement						
Unadjusted	8375 (−58)	10 750 (−57)	13 125 (−56)	15 500 (−56)	17 875 (−55)	20 250 (−55)
Quality of life–adjusted^b^	9306 (−67)	11 944 (−67)	14 583 (−66)	17 222 (−66)	19 861 (−65)	22 500 (−65)
$15 000 per patient Safer@Home reimbursement						
Unadjusted	23 375 (17)	25 750 (3)	28 125 (−6)	30 500 (−13)	32 875 (−18)	35 250 (−22)
Quality of life–adjusted^b^	25 972 (−9)	28 611 (−20)	31 250 (−27)	33 889 (−32)	36 528 (−36)	39 167 (−39)
Per patient Safer@Home reimbursement (% of cost) to achieve payer cost neutrality						
Unadjusted	11 625 (58)	14 250 (57)	16 875 (56)	19 500 (56)	22 125 (55)	24 750 (55)
Quality of–life adjusted^b^	19 266 (67)	$23 770 (67)	$28 274 (66)	32 778 (66)	37 282 (65)	41 786 (65)

^a^ Base case presumed Safer@Home enabled 75% reduction in hospital billing and 30% reduction in lost wages and out of pocket costs.

^b^ Quality adjustment assumes hospitalized patients’ quality of life score of 0.7, and discharged home on Safer@Home quality of life score of 0.9 (where 1 = normal, 0.6 = debilitated from critical illness, and 0 = death).^21,22,23,24^ Quality adjustment applies to both the costs without the program (divided by 0.7) and with the program (divided by 0.9).

We modeled the payer cost impact of a $15 000 per case reimbursement (one-half the base case estimated cost per case). With such a reimbursement, the program remained cost-saving to payers as long as payer costs per hospitalization without the program were more than $25 000 without adjusting for QoL. With QoL adjustment, all outcomes were cost-saving (Table 3). We also calculated cost reimbursement thresholds that resulted in neutral costs to payers, and found that if reimbursement was 55% to 58% of hospital costs without QoL adjustment, or up to 67% with QoL adjustment of hospital costs, the program remained cost-saving or neutral to payers (Table 3).

Finally, a sensitivity analysis modeling lower (60% of hospital costs and 15% of lost wages) or higher (90% of hospital costs and 45% of lost wages) costs demonstrated that reimbursing the program at approximately 40% to 55% or 70% to 75% of inpatient costs, depending on QoL adjustment or not, enabled payer cost neutrality (Table 4).

**Table 4.** Sensitivity Analysis for Reimbursement Threshold to Achieve Payer Cost Neutrality

Assumption	Per patient Safer@Home reimbursement (% of cost per case) to achieve payer cost neutrality by breakdown of total payer cost per case without Safer@Home (hospital billing and lost productivity), $ (%)					
	$20 000	$25 000	$30 000	$35 000	$40 000	$45 000
Assuming program achieves 60% reduction hospital billing and 15% reduction in lost wages						
Unadjusted	8625 (43)	$10 500 (42)	12 375 (41)	14 250 (41)	16 125 (40)	18 000 (40)
Quality of life–adjusted^a^						
0.7 vs 0.9	15 933 (56)	19 603 (55)	23 274 (54)	26 944 (54)	30 615 (54)	34 286 (53)
0.75 vs 0.85	13 284 (50)	16 275 (49)	19 265 (48)	22 255 (48)	25 245 (47)	28 235 (47)
Assuming the program achieves 90% reduction hospital billing, 45% reduction in lost wages						
Unadjusted	14 625 (73)	18 000 (72)	21 375 (71)	24 750 (71)	28 125 (70)	31 500 (70)
Quality of life–adjusted^a^						
0.7 vs 0.9	20 893 (73)	25 714 (72)	30 536 (71)	35 357 (71)	40 179 (70)	45 000 (70)
0.75 vs 0.85	20 343 (76)	25 098 (75)	29 853 (75)	34 608 (74)	39 363 (74)	44 118 (74)

^a^ Quality of life assumes hospitalized patients’ quality of life score of 0.7 or 0.75 and discharged home on Safer@Home quality of life score of 0.9 or 0.85 (where 1 = normal and 0.6 = debilitated from critical illness).^21,22,23,24^

## Discussion

In this economic evaluation study, we found that a new, all-virtual, at-home acute care model resulted in substantial hospital and payer savings at our large, public hospital in Los Angeles. However, the program is not currently reimbursed. From the hospital’s perspective, the program net lost money for patients with Medicare or commercial insurance due to lost or avoided revenue from inpatient stays, and net saved money for patients with Medicaid (due to low reimbursement for inpatient stays) or uninsured patients. As such, only a hospital with a payer mix typical of a safety net hospital would experience net financial savings from the program.

Indeed, the principal factor of hospital cost-savings or losses was the payer mix. Daily variable cost rates played a secondary role. While these findings should encourage safety net systems looking for out-of-hospital care alternatives, they should be alarming to payers and health care regulators. These results indicate that current payment structures encourage hospitalization of patients who may no longer require inpatient care due to advances in clinical medicine and likely serve as an impediment to development and implementation of innovative care models that provide more patient-centered, safe, high-value care out of the hospital.

However, in contrast with hospitals, payer savings occurred irrespective of payer mix because inpatient stays were replaced with much less expensive all-virtual homecare. Modeling demonstrated that a simple program reimbursement schema would enable cost-savings for both the hospital and payers. These findings indicate that the Safer@Home all-virtual care model is therefore not only cost-effective, but cost-reducing, while providing the extra value to the patient of enabling faster return to a home environment.

### Limitations

Our study has several limitations. Modeled variable costs were based on average inpatient costs across all patients cared for at LA General during the study period. Because our hospital does not generate line-item bills, it was not possible to link costs to individual patients. Inclusion of all patients has the potential to inflate daily average variable costs, and thus programmatic cost-savings, by including patients that were critically ill and therefore not eligible for Safer@Home enrollment. However, an advantage of this method was that the variable costs reflected actually incurred expenditures rather than hospital charges, which may poorly correlate with expenses. Another limitation that may affect generalizability is the variability by which Medicaid is paid in various states (eg, flat daily rate vs DRG-based). We also did not impute revenue for potential additional admissions that could have backfilled beds emptied by shorter lengths of stay. Our hospital’s payer mix was such that very few patients would generate revenue (15% Medicare or private insurance); even at hospitals with more paying insurance, any incremental revenue would depend on the particular diagnoses admitted and whether excess capacity already existed (ie, if the hospital already had some empty bed capacity, freeing up more beds would not increase revenue). Thus, our modeling took a conservative, worst case financial modeling approach to hospital costs. Another limitation is the potential that an all-virtual hospital-at-home program could be used to replace care that would have been delivered in an outpatient setting anyway, rather than for more efficient care that would have been inpatient without the program. We spent considerable effort to ensure that this did not happen for Safer@Home,^14^ and such effort would need to be replicated during program generalization. Payers should consider guardrails to ensure that any DRG-based payments are used to replace care that would otherwise have been inpatient.

## Conclusions

In conclusion, in this economic evaluation study of a novel, all-virtual, at-home alternative to hospitalization, we found that the program achieved significant avoidance of hospital days and associated cost savings, without compromising patient safety. The program was readily implemented in an urban, teaching, safety net hospital that cares for predominantly lower-income patients, with a high proportion of Medicaid and self-pay. Ironically, we believe our unique payer mix allowed us to develop and implement this more patient-centered, enhanced care model for our underprivileged patients because it would not be cost-feasible at hospitals that lost money from avoiding admitting insured patients. To encourage adoption elsewhere, such programs should be incentivized by payers and regulators who seek to enhance provision of high-value health care in the US and abroad.

## Notes

Supplement 1. **eTable 1.** DME Distributed and Costs for Each Diagnostic Bundle **eTable 2.** Patients With Revenue Loss **eTable 3.** Reimbursement Per Patient Necessary to Enable Net Zero Hospital Cost of Safer@Home Under Different Payor Mix and Daily Variable Cost Assumptions

Supplement 2.
Data Sharing Statement
