# Supplementary material for: Health Economic Analysis of an All-Virtual, At-Home Acute Care Model
Source: JAMA Netw Open. 2025 Jun 23;8(6):e2517114. doi: 10.1001/jamanetworkopen.2025.17114 (PMC12186509; doi:10.1001/jamanetworkopen.2025.17114)
Supplement: Supplement 1. — eTable 1. DME Distributed and Costs for Each Diagnostic Bundle eTable 2. Patients With Revenue Loss eTable 3. Reimbursement Per Patient Necessary to Enable Net Zero Hospital Cost of Safer@Home Under Different Payor Mix and Daily Variable Cost Assumptions [file jamanetwopen-e2517114-s001.pdf]

Supplemental Online Content

Spellburg B, Lynch C, Yee HF, Banerjee J. Health economic analysis of an all-virtual at-home acute care model. *JAMA Netw. Open*. 2025;8(6):e2517114. doi:10.1001/jamanetworkopen.2025.17114

eTable 1. DME Distributed and Costs for Each Diagnostic Bundle

eTable 2. Patients With Revenue Loss

eTable 3. Reimbursement Per Patient Necessary to Enable Net Zero Hospital Cost of Safer@Home Under Different Payor Mix and Daily Variable Cost Assumptions

This supplemental material has been provided by the authors to give readers additional information about their work.

© 2025 Spellburg B et al. *JAMA Network Open*.

eTable 1. DME Distributed and Costs for Each Diagnostic Bundle

DME	Cost Per Unit	Cellu-litis	DFI	Osteo	cUTI	COVID	Viral PNA	Bact PNA	Asthma	COPD	CHF	Other
**Thermometer**	$7.78	1	1	1	1	1	1	1	1	1	1	1
**Pulse Oximeter**	$30	1	1	1	1	1	1	1	1	1	1	1
**Digital Scale**	$45.52										1	
**BP Cuffs**	$2										1	
**BP Device**	$54.40										1	
**Face Mask**	$2.34								1	1		
**O_2 Concentrator**	$750					1	1	1	1	1		
**O_2 Tank**	$200					1	1	1	1	1		
**Nebulizer**	$70.17								1	1		
**Cost Per Patient**		$37.78	$37.78	$37.78	$37.78	$987.78	$987.78	$987.78	$1060.29	$1060.29	$139.70	$37.78
**# Patients**		126	299	17	205	20	7	58	24	21	46	53
**Cost Per Dx**		$4,760	$11,296	$642	$7,745	$19,776	$6,914	$57,291	$25,447	$22,266	$6,426	$2,002
**Total Cost**		**$164,547**										

© 2025 Spellburg B et al. *JAMA Network Open*.

eTable 2. Patients With Revenue Loss

Payor	N (%)	Total Lost Revenue	Mean Loss Per Case	Percentage of Revenue Lost
Medicare	78 (24.7%)	$(1,876,585)	$(24,058)	46.6%
Medicaid	224 (70.9%)	$(1,670,312)	$(7,457)	41.5%
Commercial	11 (3.5%)	$(444,152)	$(40,377)	11.0%
Tricare/VA	3 (1.0%)	$(34,256)	$(11,418)	0.9%
**Total**	**316**	**$(4,025,305)**	**$(12,738)**	

© 2025 Spellburg B et al. *JAMA Network Open*.

eTable 3. Reimbursement Per Patient Necessary to Enable Net Zero Hospital Cost of Safer@Home Under Different Payor Mix and Daily Variable Cost Assumptions

Payor Mix	Base Case Daily Variable Costs ($2,945)	Daily Variable Costs 50% Lower ($1,473)	Daily Variable Costs 25% Lower ($2,209)	Daily Variable Costs 25% Higher ($3,681)	Daily Variable Costs 50% Higher ($4,418)
**Base Case Payer Mix**	N/A	N/A	N/A	N/A	N/A
**Typical Payer Mix***	$13,198	$21,084	$17,141	$9,255	$5,312
**Half Typical Commercial & Medicare Mix**	$1,727	$12,169	$6,948	N/A	N/A

N/A = program net cost saving even without reimbursement.
*Typical payor mix as reflected in Table 2.

© 2025 Spellburg B et al. *JAMA Network Open*.
