# Supplementary material for: Health Economic Analysis of an All-Virtual, At-Home Acute Care Model
Source: JAMA Netw Open. 2025 Jun 23;8(6):e2517114. doi: 10.1001/jamanetworkopen.2025.17114 (PMC12186509; doi:10.1001/jamanetworkopen.2025.17114)
Supplement: Supplement 2. — Data Sharing Statement [file jamanetwopen-e2517114-s002.pdf]

Data Sharing Statement

Spellberg. Health Economic Analysis of an All-Virtual At-Home Acute Care Model. *JAMA Netw Open*. Published June 23, 2025. doi:10.1001/jamanetworkopen.2025.17114

Data
Data available: No

Additional Information
Explanation for why data not available: This was a QI project, informed consent waiver so cannot release PHI
